# Excitatory Amino Acid Transporter EAAT5 Improves Temporal Resolution in the Retina

**DOI:** 10.1523/ENEURO.0406-21.2021

**Published:** 2021-12-07

**Authors:** Jana Gehlen, Christoph Aretzweiler, Anja Mataruga, Christoph Fahlke, Frank Müller

**Affiliations:** Molecular and Cellular Physiology (IBI-1), Institute of Biological Information Processing, Forschungszentrum Jülich GmbH, D-52428 Jülich, Germany

**Keywords:** EAAT5, glutamate transporter, retina

## Abstract

Excitatory amino acid transporters (EAATs) remove glutamate from the synaptic cleft. In the retina, EAAT1 and EAAT2 are considered the major glutamate transporters. However, it has not yet been possible to determine how EAAT5 shapes the retinal light responses because of the lack of a selective EAAT5 blocker or EAAT5 knock-out (KO) animal model. In this study, EAAT5 was found to be expressed in a punctate manner close to release sites of glutamatergic synapses in the mouse retina. Light responses from retinae of wild-type (WT) and of a newly generated model with a targeted deletion of EAAT5 (EAAT5^−/−^) were recorded *in vitro* using multielectrode arrays (MEAs). Flicker resolution was considerably lower in EAAT5^−/−^ retinae than in WT retinae. The close proximity to the glutamate release site makes EAAT5 an ideal tool to improve temporal information processing in the retina by controlling information transfer at glutamatergic synapses.

## Significance Statement

Neurons communicate with other neurons at synaptic connections by release of neurotransmitters acting at postsynaptic receptors. Neurotransmitters are removed from the synaptic cleft by transporters. Using the mouse retina as a model for the central nervous system, the role of Excitatory amino acid transporter (EAAT)5 that functions as glutamate transporter and as glutamate-gated ion channel was investigated in retinal information processing. EAAT5 was found highly localized to the glutamate release site at retinal synapses, suggesting a role in shaping of synaptic responses. In a mouse model devoid of EAAT5, temporal resolution of the retina was severely compromised. The results demonstrate that glutamate transporters like EAAT5 can exert a tremendous effect on information processing in neuronal networks.

## Introduction

Excitatory amino acid transporters (EAATs) are secondary active transporters that belong to solute carrier family 1 (SLC1). EAATs rapidly remove glutamate from the synaptic cleft after its release from presynaptic nerve terminals (Vandenberg and Ryan, 2013; Rose et al., 2018) to terminate glutamatergic synaptic transmission and prevent glutamate excitotoxicity. EAATs function as both glutamate transporters and glutamate-gated anion channels (Machtens et al., 2015; Fahlke et al., 2016). To date, five different EAAT isoforms have been described in mammals (EAAT1–EAAT5). These differ significantly in their glutamate transport rates (Mim et al., 2005; Gameiro et al., 2011), but not in their unitary anion current amplitudes (Torres-Salazar and Fahlke, 2007; Schneider et al., 2014). Whereas EAATs 1–3 are effective glutamate transporters (with glutamate uptake currents that exceed their anion currents), the transport rates of EAAT4 and EAAT5 are smaller; therefore, they are assumed to predominantly function as glutamate-gated anion channels.

Glutamate is the major excitatory neurotransmitter in the mammalian retina, where it is used by photoreceptors in the outer plexiform layer (OPL) and bipolar cells in the inner plexiform layer (IPL). EAAT1 (also called GLAST in the mouse) is expressed at high levels in Müller cells and seems to be responsible for most retinal glutamate reuptake (Derouiche and Rauen, 1995; Rauen et al., 1996, 1997; Lehre et al., 1997; Pow and Barnett, 1999; Izumi et al., 2002; Kugler and Beyer, 2003; Fyk-Kolodziej et al., 2004; Sarthy et al., 2005). EAAT2 (GLT-1) is expressed in photoreceptors and bipolar cells (Rauen et al., 1996, 2004; Harada et al., 1998; both glutamatergic cell types), suggesting a role for EAAT2 in glutamate reuptake and recycling. The retinal distribution and function of EAAT5 are less clear owing to lack of a selective EAAT5 blocker or an EAAT5 knock-out (KO) animal model. EAAT5 has been localized immunohistochemically to both synaptic layers (Fyk-Kolodziej et al., 2004; Wersinger et al., 2006; Tse et al., 2014), but also to the somata of some bipolar, amacrine, and ganglion cells, as well as to photoreceptors, including their inner segments (Pow and Barnett, 2000; Wersinger et al., 2006; Lee et al., 2013). Electrophysiological measurements have also suggested the presence of EAAT5 on the terminals of photoreceptors and rod bipolar cells (Wersinger et al., 2006; Veruki et al., 2006).

In this study, the first genetically modified mouse model (EAAT5^−/−^) with a targeted deletion of the *Slc1a7* gene, which encodes EAAT5 was generated and analyzed. The EAAT5 expression pattern was defined in normal mouse retinae using a newly generated monoclonal anti-EAAT5 antibody. EAAT5 was strongly expressed in a punctate manner close to glutamate release sites in both synaptic layers of the mouse retina. This signal was completely lost in the EAAT5^−/−^ retina, confirming the specificity of the antibody. The EAAT5^−/−^ retina displays normal anatomic and synaptic organization, and features robust light responses in the form of the local field potential (LFP) or ganglion cell spiking under all light regimes. However, flicker resolution was considerably compromised in EAAT5^−/−^ retina, suggesting that an important role of EAAT5 is to improve temporal resolution in the retina.

## Materials and Methods

### Animals

Wild-type (WT) C57BL/6J and C57Bl/6N mice were obtained from Charles River. C57Bl/6N-EAAT5^−/−^ mice were generated by microinjection of the transcription activator-like effector nuclease (TALEN) in fertilized eggs (Cyagen Biosciences). Breeding animals delivered by the company displayed irregular retinal morphology that was observed in the +/+, +/−, and −/− genotypes. Mice were back-crossed into the C57Bl/6J background for nine generations. The resulting mice had normal retinal morphology, and immunohistochemical staining with a variety of retinal markers yielded results identical to WT strains. C57Bl/6J-EAAT5^−/−^ and C57Bl/6J-EAAT5^+/+^ mice from heterozygous breeding were used for electrophysiological measurements and immunohistochemistry studies. All animals were kept on a 12 h light/dark cycle with food and water *ad libitum*. Zeitgeber time (ZT) of the experiments was 3–8 (ZT0 = 7 A.M.) All experiments were performed in accordance with the German Law for the Protection of Animals and following approval by the regulatory authorities.

### Generation of the anti-EAAT5 antibody, 20F12

A monoclonal antibody against murine EAAT5 was generated using a glutathione *S*-transferase (GST) fusion protein. An intracellular epitope (C-terminal amino acids 441–560) present in all postulated splice variants of rat EAAT5 (Lee et al., 2013) was chosen to ensure the detection of all potential murine variants. The amino acid sequence was: tddinliiavdwaldrfrtminvlgdalaagimahicrkdfaqdmgtekllpcetkpvtlqeivaaqqngcvksvaeaseltlgptcphhipvqveqdedpaaasldhctieiseletnv.

Rats (strain: Lou/C) were immunized with the GST-fusion construct using standard procedures (R. Feederle, Antibody Core Facility, Helmholtz Zentrum München), and spleen cells were fused to a mouse myeloma cell line (P3X63-Ag8.653) to generate hybridoma cells. The primary supernatant was tested by ELISA and then immunochemically. Hybridoma cells were subcloned via single-cell distribution until all reacted positively. The final clone was expanded and used to generate supernatant containing the monoclonal antibody (20F12; subtype IgG_2_).

### Immunohistochemistry

#### Sections

For immunohistochemistry, animals were deeply anesthetized with isoflurane and killed by decapitation. The eyes were then enucleated and opened by an encircling cut at the limbus. Eyecups with the retinae were immersion-fixed for 30 min in 4% paraformaldehyde in 0.1 m phosphate buffer, pH 7.4 (PB) at room temperature (RT) and then washed in PB several times. The tissue was incubated in 10% sucrose in PB for 1 h, and then in 30% sucrose in PB overnight. Retinae were then isolated from the eyecups, flat embedded and frozen in optimal cutting temperature compound (NEG-50, Richard Allen Scientific, Thermo Fisher Scientific). Vertical sections (i.e., perpendicular to the retinal layers, 20-μm thick) were cut on a cryostat (HM 560 CryoStat, Microm), collected on SuperFrost Plus slides (Menzel), and stored at −20°C.

#### Dissociated cells

Eyes were prepared as described above. Retinae of one animal were removed from the eyecups, washed in 1 ml warm calcium/magnesium-free HBSS (CMF-HBSS; Sigma Aldrich) for 5 min at 37°C, and incubated for 20 min at 37°C in 1-ml warm papain (Worthington) solution (20 units of papain in 1-ml CMF-HBSS). Papain action was stopped by washing the retinae twice in warm HBSS. The tissue was gently dissociated in 1 ml fresh HBSS by pipetting 15–20 times using a cut and fire-polished 1000-μl plastic tip (Nerbe plus). After dissociation, cells were seeded and settled for 30 min on poly-L-lysine-coated (Sigma) coverslips at RT in a humidified chamber before fixation (5 min at RT with 4% paraformaldehyde). The fixative was then removed and cells were washed in PB (twice for 10 min each).

#### Antibody staining

For anti-EAAT5 antibody (20F12) staining, an antigen retrieval step was performed: 5-min pretreatment with 1% SDS (AppliChem) in phosphate-buffered saline, followed by three 5-min washes in PB at RT. Sections were incubated overnight at RT with primary antibody diluted in 5% Chemiblocker (Chemicon), 0.5% Triton X-100, and 0.05% NaN_3_ in PB. Sections were washed in PB, incubated for 1 h at RT with fluorescently labeled secondary antibody diluted in PB containing 5% Chemiblocker and 0.5% Triton X-100, and then washed in PB. The 20F12 antibody (rat IgG_2_ subtype) was detected with secondary mouse anti-rat-IgG2 antibody (gift of R. Feederle, Helmholtz Zentrum München; 1:100 in CTA; 1 h at RT) and visualized with donkey-anti-mouse Cy3 antibody. Retinal sections were then mounted on coverslips using Aqua-Poly/Mount (Polysciences). Sections and cells were examined using a confocal laser scanning microscope (Leica TCS SP5, Leica Microsystems) with a 63×/1.4 oil immersion lens. Images were processed using Adobe Photoshop.

Primary antibodies are listed in Table 1.

**Table 1:** Primary antibodies and markers

Antibody name, antigen	Host	Dilution	Source	RRID
20F12 against EAAT5	Rat	1:500	Anja Mataruga, Forschungszentrum Jülich and R. Feederle, Helmholtz Zentrum München, Germany	None
EAAT5	Goat	1:100	Santa Cruz, Sc-18779	AB_2190894
EAAT1	Mouse	1:500	Miltenyi Biotech, MACS 130-095-822	AB_10829302
EAAT2	Rat	1:2000	D. Pow, Department of Physiology and Pharmacology, University of Queensland, Australia	None
mGluR6	Rabbit	1:7500	S. Nakanishi, Department of Biologicalal Sciences, Kyoto University, Japan	None
PKCα	Mouse	1:500	Santa Cruz, sc208	AB_2168668
Piccolo	Guinea pig	1:500	Synaptic Systems 142104	AB_2619831
vGlut1	Guinea pig	1:30,000	Chemicon, ab5905	AB_2301751
Recoverin	Rabbit	1:2000	Chemicon, ab5585	AB_2253622
PSD95	Mouse	1:200	Sigma, p246	AB_260911
7D8 against cone cyclic nucleotide-gated channel	Rat	1:50	Anja Mataruga, Forschungszentrum Jülich and E. Kremmer, Helmholtz Zentrum München, Germany	None
1D4 against rhodopsin	Mouse	1:500	B. Molday University of British Columbia, Vancouver, Canada	None
GFAP	Chicken	1:2000	Novus, nb110–58368	AB_921444
Glutamine synthetase	Rat	1:2000	BD Biosciences, 610517	AB_397879
CaBP	Mouse	1:400	Sigma, C-8666	AB_2313712
Calretinin	Goat	1:3000	Chemicon, ab1550	AB_90764
ChAT	Mouse	1:400	Millipore, MAB5270	AB_2079753
CTBP2	mouse	1:200	Abcam, c33020	None
CaV pan calcium channel α1 subunit	Rabbit	1:200	Alomone Labs, ACC-004	AB_2039762
Biotinylated peanut agglutinin	n.a.	1:1600	Sigma, 16135	None

Secondary antibodies are listed in Table 2. Streptavidin Alexa Fluor 647 (Invitrogen), at 1:100 dilution, was used to visualize biotinylated peanut agglutinin.

**Table 2:** Secondary antibodies and markers

Antibody	Host	Dilution	Source
Anti-rabbit Cy2	Donkey	1:400	Dianova
Anti-rabbit Cy3	Donkey	1:500	Dianova
Anti-rabbit Cy5	Donkey	1:500	Dianova
Anti-mouse Cy2	Donkey	1:100	Dianova
Anti-mouse Cy3	Donkey	1:100	Dianova
Anti-mouse Cy5	Donkey	1:200	Dianova
Anti-chicken Cy2	Donkey	1:200	Dianova
Anti-rat Cy3	Donkey	1:500	Dianova
Anti-guinea pig Dy649	Donkey	1:500	Dianova
Anti-guinea pig Cy2	Donkey	1:400	Dianova
Anti-goat Cy3	Donkey	1:1000	Dianova
Anti-goat A647	Donkey	1:200	Dianova
Streptavidin Alexa Fluor 647	n.a.	1:100	Molecular Probes

### Multielectrode arrays (MEAs) and data recording

MEAs (Multi Channel Systems MCS GmbH) had 60 active electrodes in an 8 × 8 matrix layout with electrode diameters (ø) of 30 μm and interelectrode distances of 200 μm. Electrodes were coated with porous titanium nitride with impedance levels of 50 kΩ at 1 kHz. MEAs were pretreated in a plasma cleaner (Diener Electronic GmbH + Co KG) and coated with poly-d-lysine hydrobromide (Sigma).

The MEA60 data acquisition system (MC_Card, Multichannel System) consisted of an RS-232 interface, an integrated preamplifier and MEA 1060 bandpass filter (amplification gain: 1200), and a personal computer. The waveforms were recorded with a sampling frequency rate of 25 kHz/channel. Data were later converted to ASCII files by MC_Data for further analysis with OriginPro8 and custom-made MATLAB scripts.

Retinae of adult WT and EAAT5^−/−^ mice were prepared for MEA recordings. Briefly, mice were dark-adapted overnight. Retina preparation and recording were performed in dim red light. Mice were deeply anesthetized with isoflurane and killed by decapitation. The eyeballs were enucleated and retinae were isolated in carbonate-buffered Ames’ solution (Sigma), bubbled with 95% O_2_ + 5% CO_2_ at a pH of ∼7.4 (AMES). Retinae were cut in half and stored in AMES in the dark at RT. For the experiment, one half retina was transferred with the ganglion cell side up onto a nitrocellulose filter (pore size 0.8 μm, MF-Millipore Membrane Filters, Millipore, size 5 × 5 mm with a central hole of 2-mm diameter). The filter/retina sandwich was transferred onto an inverted Petri dish, excess buffer was removed with filter paper, and the sandwich was mounted with the ganglion cells toward the electrode side of the MEA. During recording, the retina was continuously perfused with AMES at a flow rate of 3 ml/min at RT.

#### Light stimulation and analysis

Retinae were stimulated in full-field mode using a white LED (ANSI white, 3465K, 185 lm at 700 mA, rise and fall time of ∼100 μs) positioned below the MEA. Stimuli were generated by an external stimulator (STG 4002, Multi Channel Systems MCS GmbH) controlled by MC-Stimulus software. Each light flash was 20 ms in length. Flicker stimuli were either applied with increasing frequency at a single light intensity or at a single frequency with rising intensity. Intensities were calculated as rhodopsin isomerizations per rod and flash (rhod*/rod/flash), according to Wyszecki and Stiles (2000) and Lyubarsky et al. (2004). Scotopic and mesopic stimuli were applied without background light. For photopic stimulation, the retina was light-adapted on the MEA by a background light of 10,000 rhod*/rod/s for 10 min (Seeliger et al., 2011) and photopic stimuli were applied on that background. Recordings of both LFP and action potentials were subjected to Fast Fourier Analysis using scripts in Neuroexplorer or MATLAB. The response was considered correct when the dominant peak in the FFT matched the stimulus frequency. The fraction of correct responses (normalized to the response at 2 Hz) were plotted against stimulus frequencies.

## Results

### Targeted EAAT5 deletion does not affect retinal anatomy or synaptic organization

**Figure 1.**
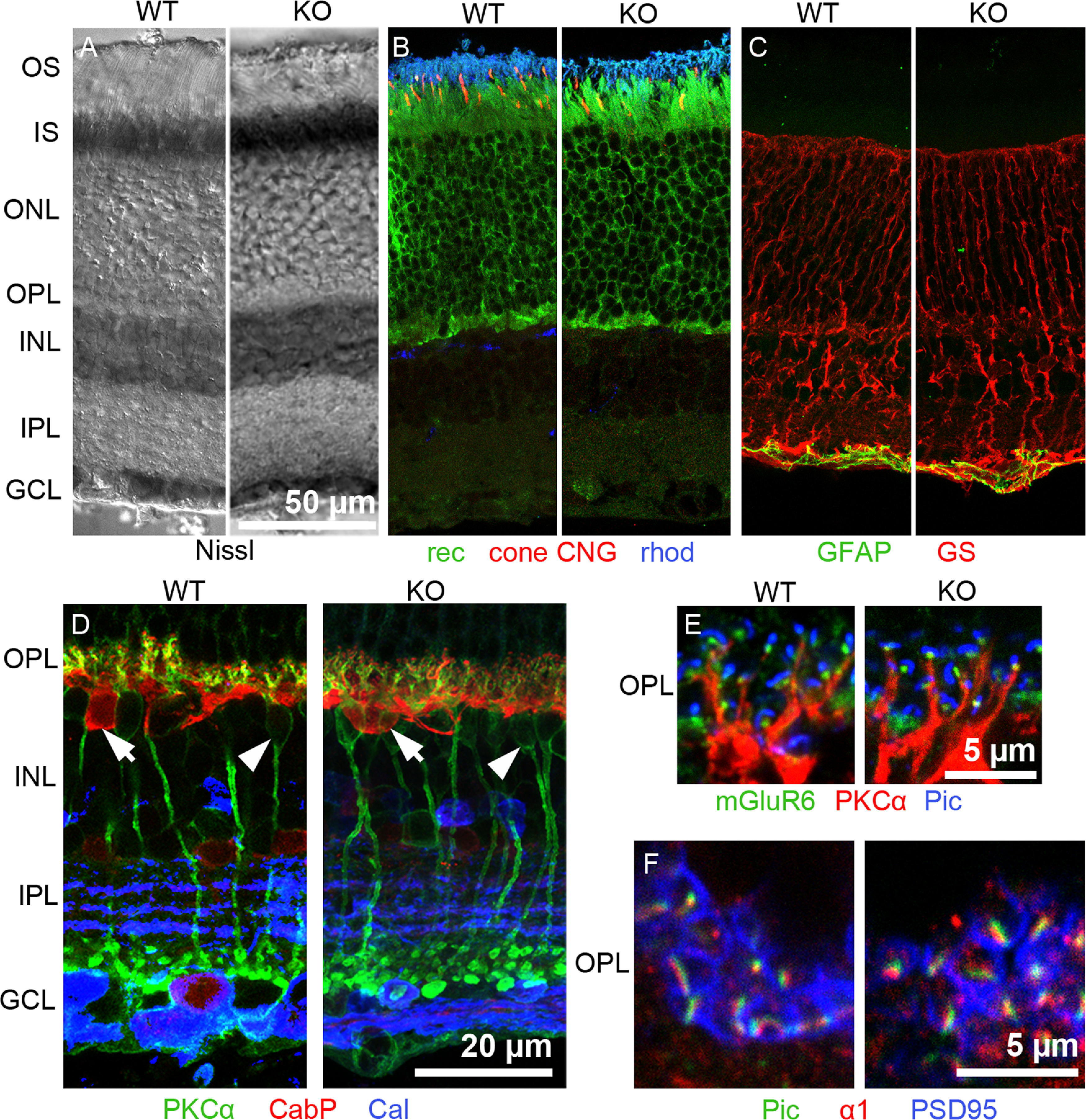
The EAAT5^−/−^ retina appears histologically normal. ***A–F***, left, WT retina (WT), right: EAAT5^−/−^ retina (KO). ***A***, Nissl staining reveals retinal thickness and retinal layering. ***B***, Photoreceptors, recoverin (rec; green, photoreceptor somata and inner segments), cone cyclic nucleotide-gated (CNG) channel (red, cone outer segments), rhodopsin (rhod; blue, rod outer segments). ***C***, Glial cells, glutamine synthetase (GS; red, Müller cells), glial fibrillary acidic protein (GFAP; green, astrocytes). ***D***, Inner retinal cells, PKCα (green, rod bipolar cells, arrowheads), CabP (red, horizontal cells, arrows), calretinin (Cal; blue, several types of amacrine cells). ***E***, OPL, mGluR6 (green, glutamate receptor on ON-bipolar cell dendrites), PKCα (red, rod bipolar cell dendrites), piccolo (Pic; blue, photoreceptor presynaptic ribbon). ***F***, OPL, piccolo (Pic; green, photoreceptor presynaptic ribbon), pan α1 calcium channel subunit (red, calcium channel in rod photoreceptors), postsynaptic density protein 95 (PSD95; blue, plasma membrane marker for rod terminals). GCL: ganglion cell layer, INL: inner nuclear layer, IPL: inner plexiform layer, IS: inner segments, ONL: outer nuclear layer, OPL: outer plexiform layer, OS: outer segments. Scale bar in ***A*** also applies to ***B***, ***C***. WT and EAAT5^−/−^: *n* = 6 animals.

Mice carrying a homozygous deletion of *Slc1a7* developed normally and were viable. They were fertile with normal mating efficiency and no apparent behavioral deficit or disorder. No anatomic differences were observed between WT (Fig. 1, WT, left) and EAAT5^−/−^ retinae (KO, right) in retinal thickness or layering (Fig. 1*A*). In addition, immunohistochemical staining patterns were similar between genotypes for markers (for details, see legend to Fig. 1) of photoreceptors (Fig. 1*B*), glia cells (Fig. 1*C*), neurons of the inner retinal network (Fig. 1*D*), and presynaptic and postsynaptic elements of photoreceptor synapses (Fig. 1*E*,*F*).

### EAAT5 is expressed in a punctate manner at glutamatergic synapses

Although no EAAT5 splice variants have been reported in the mouse, several have been described in the rat (Lee et al., 2013). Therefore, a monoclonal antibody was raised against mouse EAAT5 using a C-terminal epitope homologous to a region common to all rat EAAT5 splice variants to ensure broad specificity for all potential EAAT5 splice forms in the mouse.

**Figure 2.**
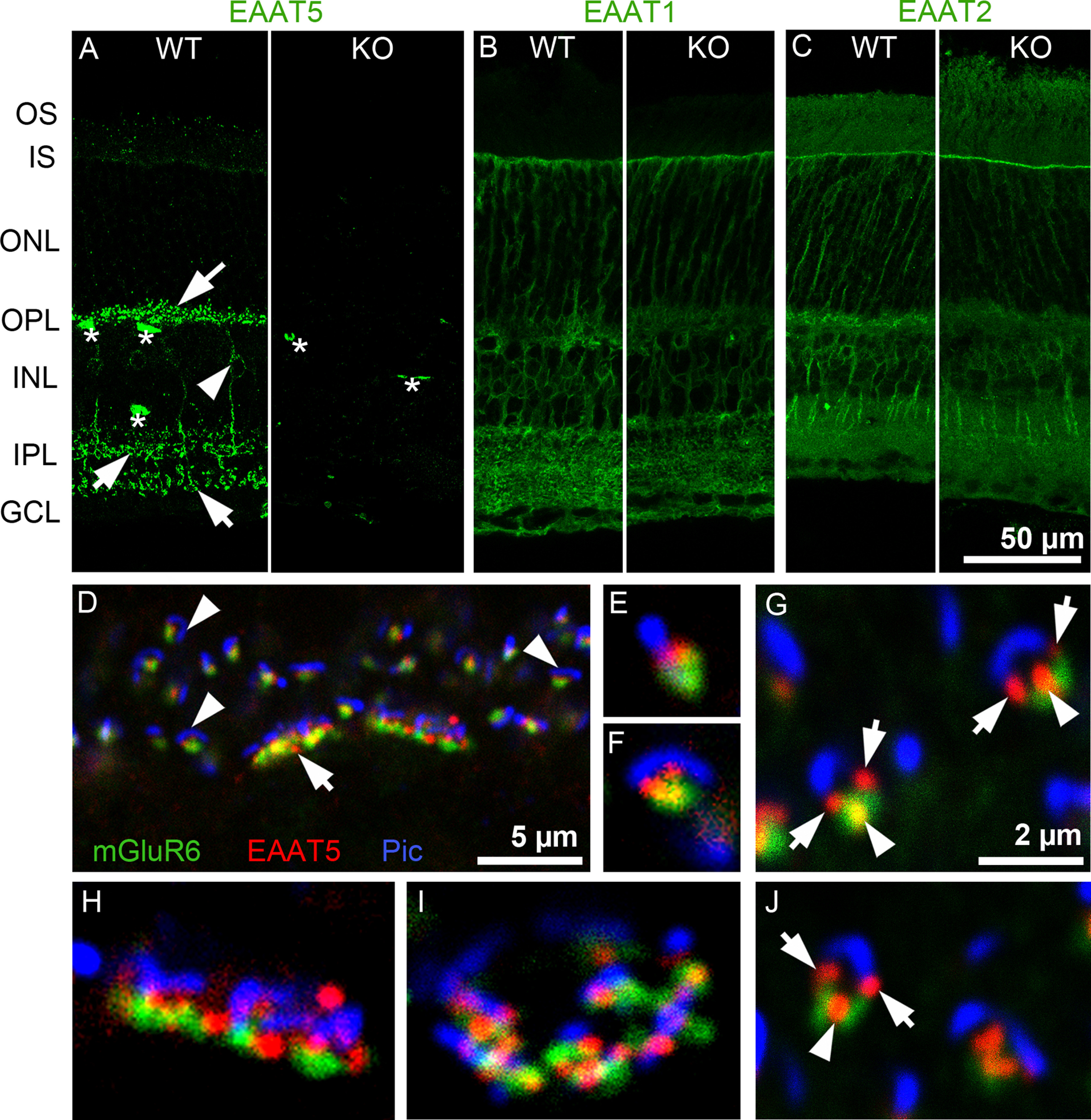
Localization of EAATs in mouse retinal sections. ***A***, EAAT5 expression. In WT retina (left), arrows indicate brightly labeled puncta or short processes in the OPL and IPL, the arrowhead indicates bipolar cell soma in the INL, and asterisks indicate nonspecific staining of blood vessels by the secondary antibody (*n* = 10 animals). In EAAT5^−/−^ (KO) retina (right), puncta are absent; only nonspecific staining in blood vessels is seen (*n* = 5 animals). ***B***, EAAT1 (GLAST) was strongly expressed in Müller cells that span almost all retinal layers. No difference in expression pattern or level was observed in the EAAT5^−/−^ retina (right; *n* = 3 animals for WT and for EAAT5^−/−^). ***C***, EAAT2 (GLT1) was mainly expressed in photoreceptors and bipolar cells, with no differences in expression between WT (left) and EAAT5^−/−^ (right) retinae (*n* = 3 animals for WT and for EAAT5^−/−^). ***D–J***, Triple staining with antibodies against mGluR6, EAAT5, and piccolo (Pic) in a WT retina (*n* = 5 animals). ***D***, In the OPL, EAAT5 (red) is closely associated with both the piccolo-positive ribbon (blue) of photoreceptors, and the mGluR6 label on ON-bipolar cell dendrites (green). Arrow, cone terminal; arrowheads, rod terminals. ***E–G***, ***J***, Higher magnification of rod spherules at different viewing angles, showing EAAT5-positive puncta (red) marked with arrows between the ribbon (blue) and mGluR6 (green) and marked with arrowheads between mGluR6 puncta. ***H***, ***I***, Single cone pedicles, showing close association of EAAT5-positive puncta with both ribbons and mGluR6. GCL: ganglion cell layer, INL: inner nuclear layer, IPL: inner plexiform layer, IS: inner segments, ONL: outer nuclear layer, OPL: outer plexiform layer, OS: outer segments. The scale bar in ***C*** also applies to ***A***, ***B***; the scale bar in ***G*** also applies to ***E***, ***F***, ***H–J***.

EAAT5 was highly localized in WT retinae, often to small puncta in the IPL and OPL (Fig. 2*A*, arrows). In the inner nuclear layer, some weakly labeled bipolar cell somata (arrowhead) were occasionally visible, with their processes projecting into the IPL. In Figure 2*A*, asterisks mark blood vessels that were nonspecifically labeled by secondary antibodies (the signal was also present in negative controls; data not shown). Importantly, punctate staining was only observed in WT but not in EAAT5^−/−^ retinae, indicating the high specificity of the antibody (Fig. 2*A*, right).

Genetic ablation of EAAT5 did not change the expression patterns of EAAT1 (GLAST; Fig. 2*B*) or EAAT2 (GLT-1; Fig. 2*C*). Consistent with previous studies in the rat (Derouiche and Rauen, 1995; Rauen et al., 1996), strong EAAT1 staining was found in Müller cell somata and their processes spanning the retina vertically. EAAT2 staining was localized to the somata and processes of photoreceptors (mostly cones) and bipolar cells (Rauen et al., 1996; Harada et al., 1998).

Figure 2*D–J* shows the close association of EAAT5-positive puncta with the glutamate release site on photoreceptors. Photoreceptor terminals comprise one (rod spherule) or several (cone pedicle) invaginations, each with a ribbon structure marking the glutamate release site and three to four postsynaptic processes formed by the dendrites of ON-bipolar and horizontal cells (Dowling and Boycott, 1966). Triple labeling was performed of EAAT5, metabotropic glutamate receptor 6 (mGluR6; the glutamate receptor expressed postsynaptically in ON-bipolar cell dendrites; Nomura et al., 1994), and the presynaptic protein piccolo. In the OPL, the anti-piccolo antibody labeled the ribbons of rods and cones (Regus-Leidig et al., 2013). In Figure 2*D*, several rod spherules (arrowheads) and two cone pedicles (arrow) could be distinguished. Figure 2*E–J* show the terminals at higher magnification. Depending on the viewing angle, the rod ribbon (blue) appeared as a line, a horseshoe shape, or an intermediate shape. The number of EAAT5 puncta per spherule seemed to depend on the viewing angle, with one punctum (Fig. 2*E*) or two puncta (Fig. 2*F*) between the mGluR6 and piccolo labels (Fig. 2*G*,*J*, arrows) or with an additional third punctum between the two mGluR6 puncta on the rod bipolar cell dendrites, these sometimes overlapped slightly but never clearly colocalized (Fig. 2*G*,*J*, arrowheads).

Multiple EAAT5 puncta might indicate EAAT5 expression by different cell types. First, the puncta might represent the tips of horizontal cells (Dowling and Boycott, 1966). However, in double labeling experiments with antibodies against the calcium-binding protein (CabP)28K (a good marker for horizontal cell dendritic tips; Haverkamp and Wässle, 2000), no colocalization with EAAT5 was observed (data not shown). Second, EAAT5 might localize to a more distal site than mGluR6 on ON-bipolar cell dendrites; however, colocalization with typical rod bipolar cell markers such as protein kinase C (PKC)α or Gα_0_ was not observed. Finally, EAAT5 might be expressed presynaptically in the photoreceptor terminal, with its localization restricted to parts of the plasma membrane within the invagination. Evidence for EAAT5 expression in photoreceptors comes from other studies (Eliasof and Werblin, 1993; Picaud et al., 1995; Pow and Barnett, 2000; Wersinger et al., 2006; Hasegawa et al., 2006; Lee et al., 2013). Cone pedicles harbor several invaginations: Figure 2*H*,*I* shows cone pedicles in the side (Fig. 2*H*) and top (Fig. 2*I*) views. Similar to in rod spherules, EAAT5 was found closely associated (but never colocalized) with the ribbons and mGluR6.

**Figure 3.**
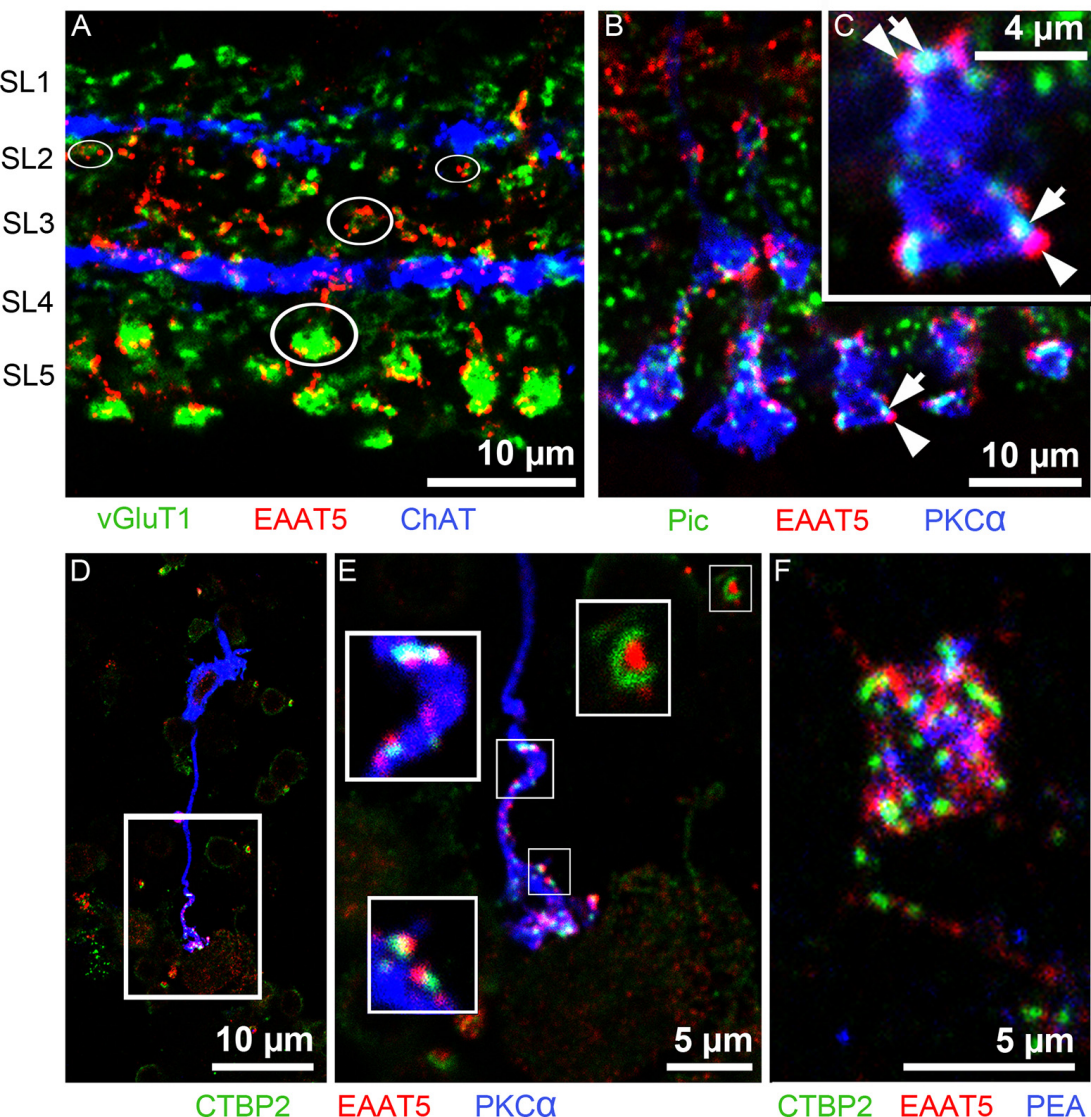
Localization of EAAT5 on bipolar cell terminals in the mouse retina. ***A***, Triple staining. EAAT5-positive puncta (red) were mostly found on vGluT1-positive (green) bipolar cell terminals in SL4 and SL5. ChAT-positive processes (blue) subdivide the IPL in a characteristic way (*n* = 3 animals). ***B***, ***C***, Triple staining. Close association of EAAT5-positive puncta (red, arrowheads) and ribbons [piccolo (Pic), green, arrows] on rod bipolar terminals (PKCα, blue; *n* = 6 animals). ***D–F***, Acutely dissociated rod bipolar cells and photoreceptor terminals (three experiments, two retinae of one animal/experiment). ***D***, Dissociated rod bipolar cell (PKCα, blue) with an axon terminal decorated with EAAT5 (red) puncta and ribbons (CTBP2, green). ***E***, Axon terminal of the same cell. Insets are at higher magnification. Top right corner, Isolated rod terminal with horseshoe-shaped ribbon (CTBP2, green) and EAAT5 puncta (red). ***F***, Isolated cone terminal [PEA (peanut agglutinin), blue] with closely associated ribbons (CTBP2 green) and EAAT5 puncta (red).

Several EAAT5-positive bands could be observed in the IPL (Fig. 2*A*). At higher magnification (Fig. 3*A*), triple labeling showed the fine detail of EAAT5 (red) localization relative to vesicular glutamate transporter 1 (vGlut1; green; labels all bipolar cell terminals; Haverkamp et al., 2003) and choline acetyltransferase (ChAT; blue; labels bands of cholinergic processes that subdivide the IPL in a characteristic manner; Haverkamp and Wässle, 2000). Decoration of bipolar cell terminals (green) with numerous EAAT5-positive puncta (white ellipses) was commonly seen in sublaminae (SL)4 and SL5 but rarer in the other SL. Most terminals in SL4 and SL5 originate from rod bipolar cells. Therefore, retinal sections were triple labeled for piccolo (green), EAAT5 (red), and PKCα (blue; to label rod bipolar cell terminals; Fig. 3*B*,*C*; Negishi et al., 1988; Greferath et al., 1990). Note that in the IPL, piccolo is also present in conventional synapses and, therefore, is seen outside bipolar cell terminals (Regus-Leidig et al., 2013). Nevertheless, ribbons of rod bipolar terminals can be easily identified by their close association with the PKC signal. As seen in photoreceptor invaginations, EAAT5 puncta were in close proximity to the glutamate release site of rod bipolar terminals marked by piccolo-positive ribbons (Fig. 3*C*, blue).

Owing to the resolution limits of light microscopy, it is difficult to unequivocally distinguish presynaptic and postsynaptic sites at a synapse in retinal sections. Therefore, cells from mouse retinae were isolated by enzymatic digestion. In the cell preparation, EAAT5 puncta were observed on the axon terminals of acutely dissociated rod bipolar cells, again in close proximity to the ribbons (Fig. 3*D*,*E*). Isolated rod and cone terminals were also found with similar EAAT5 and ribbon labeling to that seen in intact sections (compare Figs. 3*E* and 2*F* and Figs. 3*F* and 2*I*). These staining patterns suggest that EAAT5 is expressed on rod bipolar cell terminals and photoreceptor terminals, in agreement with previous studies (Fyk-Kolodziej et al., 2004; Veruki et al., 2006; Wersinger et al., 2006; Tse et al., 2014).

These results demonstrate that EAAT5 is closely associated with glutamatergic synapses in both synaptic layers of the retina, where it is perfectly located to mediate glutamate-driven negative feedback. Other than loss of the EAAT5 signal, no differences in marker staining were seen between WT and EAAT5^−/−^ retinae. Hence, targeted EAAT5 deletion does not produce gross pathologic changes in the retina. In conclusion, the EAAT5^−/−^ mouse proved a good model to study the function of EAAT5 in generating and shaping light responses in the intact retina.

### EAAT5 improves temporal resolution in the retina

**Figure 4.**
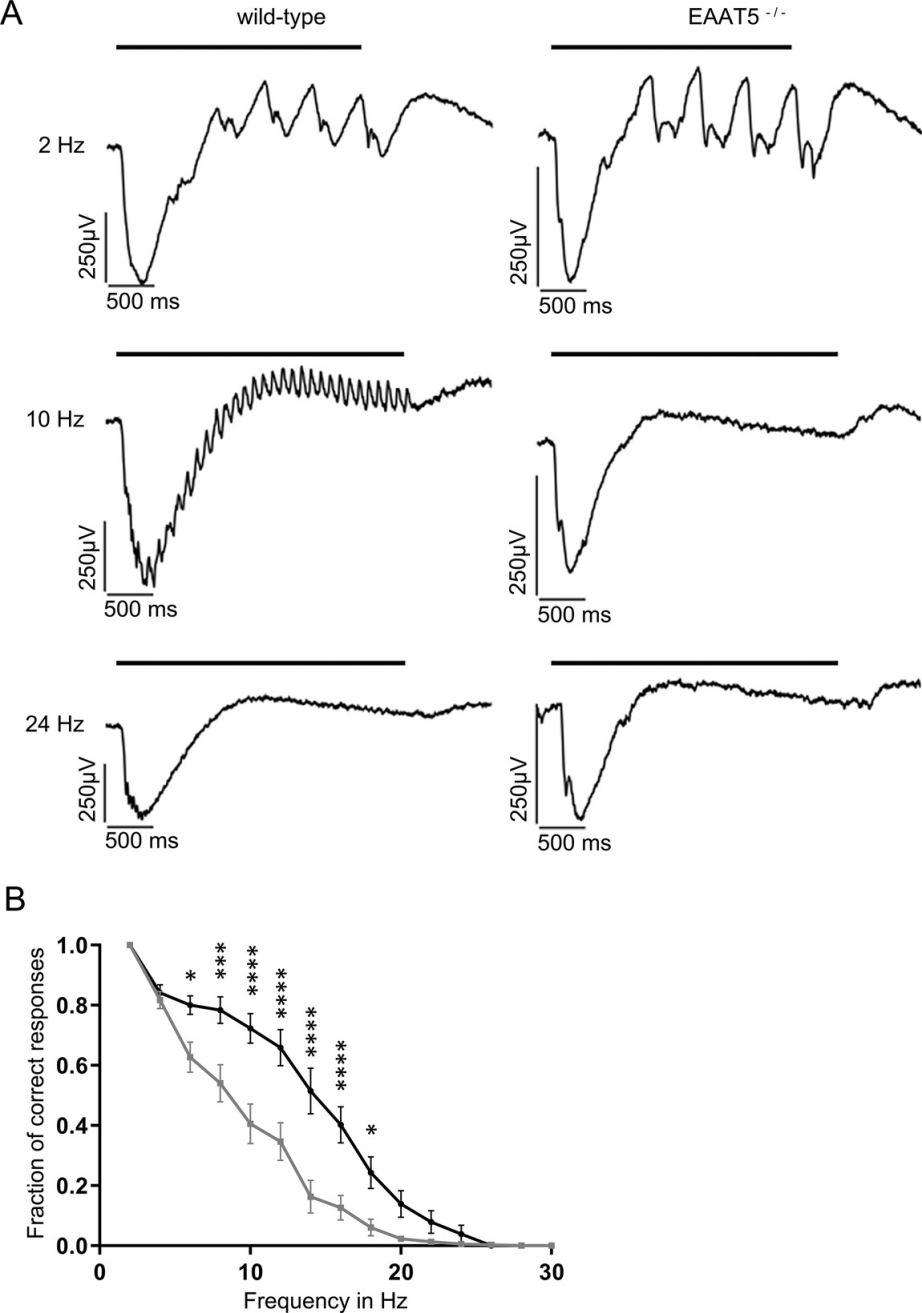
Temporal resolution of the EAAT5^−/−^ retina is significantly impaired in the mesopic range. Flicker stimuli were applied for a series of frequencies from 2 to 30 Hz. ***A***, The LFP of the EAAT5^−/−^ retina (right) showed impaired resolution for the 10-Hz flicker stimulus compared with the WT retina (left). In neither genotype could the 24-Hz flicker stimulus be resolved. Bars: duration of flicker stimulus. ***B***, When the fraction of correct responses was plotted against the stimulus frequency, a significant reduction in temporal resolution was apparent for EAAT5^−/−^ (gray curve, mean ± SEM of 30 retinal pieces from 13 animals) compared with the WT (black curve, mean ± SEM of 33 retinal pieces from 13 animals). Two-way ANOVA with Bonferroni multiple comparisons. Flicker stimulus: 610 activated rhodopsin molecules (rhod*) per rod and flash (rhod*/rod/flash); total stimulus duration: 3 s; individual flash duration: 20 ms.

Next, light responses were recorded from WT and EAAT5^−/−^ retinae using MEAs under three light regimes: scotopic (dark-adapted, low-intensity stimuli, rods only), mesopic (dark-adapted, medium-intensity stimuli, rods and cones active), and photopic (light-adapted with bright background, high-intensity stimuli, cone dominated). Flicker stimuli were applied with frequencies of between 2 Hz and 30 Hz. Depending on the light regime, responses to flicker stimuli start to fuse at different frequencies (i.e., the flicker fusion frequency, which in mouse in all lighting regimes was found to be below 30 Hz). Figure 4*A* compares representative recordings of the LFP of dark-adapted retinae from WT (left) and EAAT5^−/−^ (right) animals when stimulated in the high mesopic range. The LFP recorded *in vitro* roughly corresponds to the electroretinogram (ERG) that can be recorded *in vivo* (Fujii et al., 2016). At light onset, a negative deflection was observed that corresponds to the a-wave of the ERG (mostly originating from the closure of cyclic nucleotide-gated ion channels in photoreceptors during the photoresponse), followed by a positive deflection corresponding to the b-wave (which reflects the activity of ON-bipolar cells). In both WT and EAAT5^−/−^ retinae at 2 Hz, each successive light flash of the flicker stimulus initiated additional deflections superimposed on the overall waveform triggered by the first flash. At 24 Hz, retinae of neither genotype could resolve the flicker stimulus: both responses were consistent with the application of a continuous light stimulus. The WT retina could resolve the 10-Hz flicker stimulus, with individual responses on top of the large deflection in LFP throughout the stimulus duration, albeit at a lower amplitude than at 2 Hz. In contrast, these individual deflections could not be observed in the EAAT5^−/−^ retina (i.e., this retina could not resolve the 10-Hz flicker stimulus). For both WT and EAAT5^−/−^ retinae, the fraction of correct responses was determined and plotted over the stimulus frequency (Fig. 4*B*). In EAAT5^−/−^ retina (gray curve), temporal resolution was significantly compromised: half-maximal resolution was at 10 Hz, compared with 14 Hz in the WT retina (black curve).

**Figure 5.**
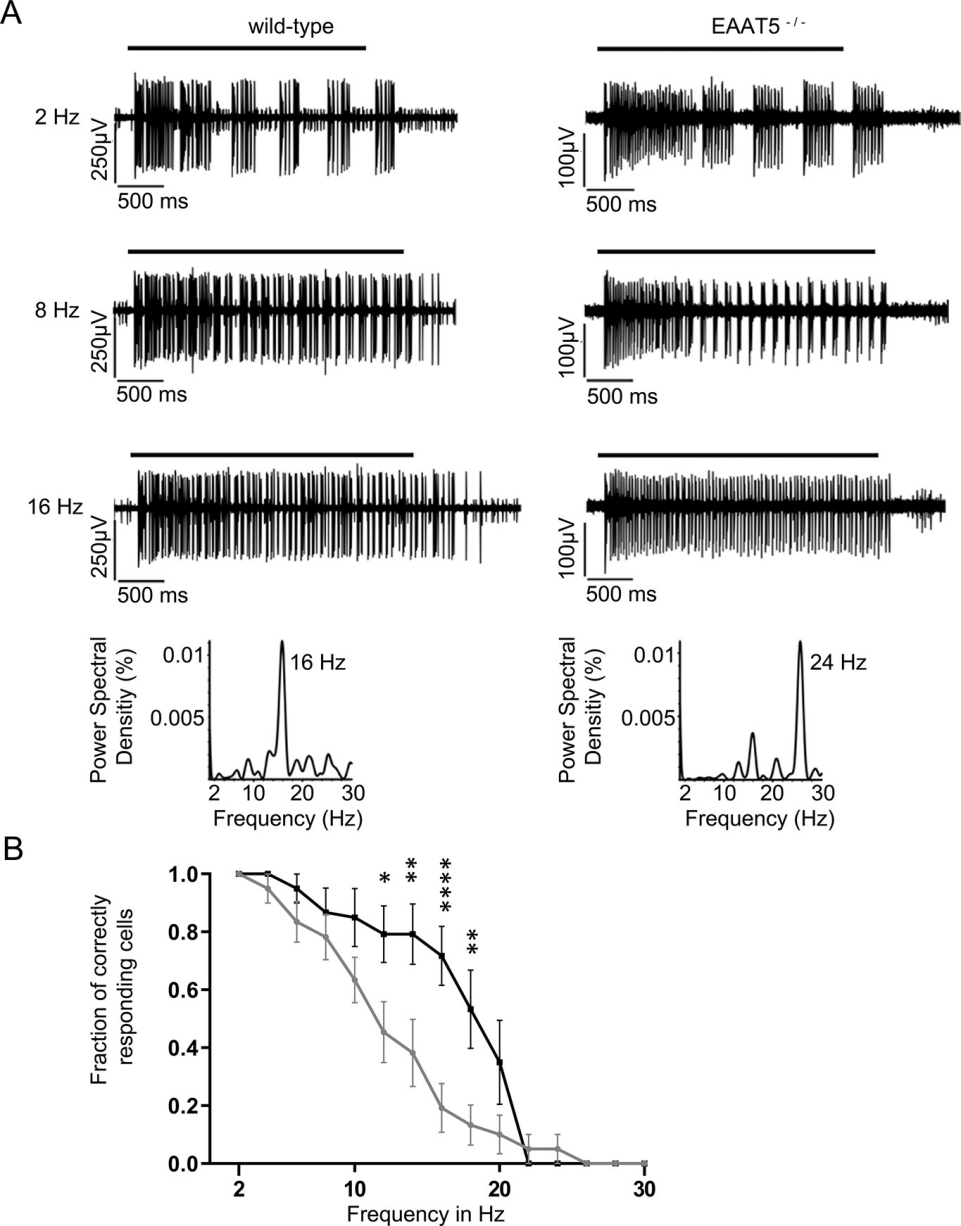
Temporal resolution of ON-ganglion cells is significantly impaired in the EAAT5^−/−^ retina in the mesopic range. ***A***, Typical response of one type of ON-ganglion cell (“ON-merge cell”) in the WT (left) and EAAT5^−/−^ retina (right). Bars: duration of flicker stimulus. ***B***, The fraction of correctly responding cells was significantly reduced in EAAT5^−/−^ (gray curve, mean ± SEM of 32 cells of 10 animals) compared with WT (black curve, mean ± SEM of 40 cells of 10 animals) cells at frequencies of 10–20 Hz. Two-way ANOVA with Bonferroni multiple comparisons. Flicker stimulus: 610 activated rhodopsin molecules (rhod*) per rod and flash (rhod*/rod/flash; mesopic conditions); total stimulus duration: 3 s; individual flash duration: 20 ms.

To test whether this difference also affects retinal output to the brain, the spiking behavior of ganglion cells was recorded. Up to 30 different types of ganglion cells are postulated in the mouse retina (Baden et al., 2016), each with a characteristic response behavior. Figure 5*A* shows one type of response that was routinely recorded in this study both in WT (left) and EAAT5^−/−^ (right) retina. In this ON-ganglion cell type, a 2-Hz flicker stimulus led to relatively long bursts triggered by the first two flashes in the first part of the response, which tended to merge (“ON-merge cell”); in the last two-thirds of the response, well-separated bursts were triggered by every flash. Flicker stimuli of 2 and 8 Hz could be resolved in both genotypes. At 16 Hz, the stimulus was well resolved in the WT cell (note the gaps separating the short bursts in the second half of the recording and the correct peak at 16 Hz in the power spectral density analysis shown below the trace) but not in the EAAT5^−/−^ cell (very few gaps and a peak at 24 Hz instead of 16 Hz). Plotting the fraction of correctly responding cells over the flicker frequency (Fig. 5*B*) revealed that temporal resolution by this cell type is significantly worse in EAAT5^−/−^ (gray curve) than WT (black curve) retina.

### The impact of EAAT5 on temporal resolution depends on stimulus intensity

**Figure 6.**
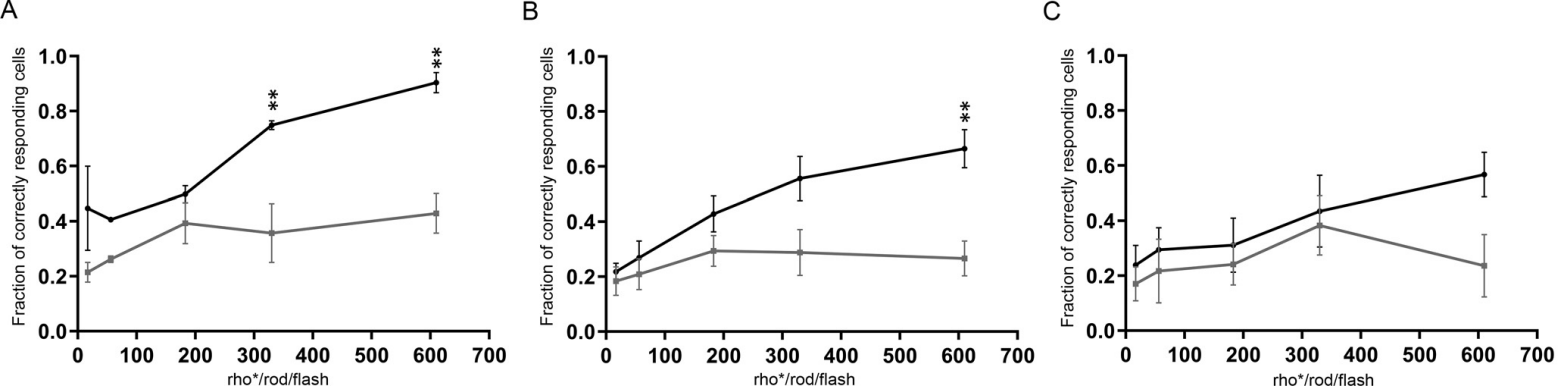
The impact of EAAT5 on temporal resolution in ganglion cells increases with stimulus intensity under mesopic conditions. Black curves: WT; gray curves: EAAT5^−/−^. ***A***, ON-merge cells (WT, 32 cells; EAAT5^−/−^ 15 cells). ***B***, ON-cells (except for ON-merge; WT, 92 cells; EAAT5^−/−^ 96 cells). ***C***, OFF-cells (WT, 39 cells; EAAT5^−/−^, 20 cells). Two-way ANOVA with Bonferroni multiple comparisons. Flicker stimulus: 12 Hz, total stimulus duration: 3 s; individual flash duration: 20 ms. Stimulus intensities: 17, 56, 183, 330, 610 activated rhodopsin molecules (rhod*) per rod and flash (rhod*/rod/flash).

The bright light stimuli employed in these experiments (Figs. 4, 5) resulted in large changes in the membrane potential of photoreceptors and bipolar cells concomitant with large changes in the amount of glutamate released at the terminals of these cells. It is reasonable to assume that the impact of EAAT5 on the shaping of retinal light responses depends on the extent to which glutamate release is changed by the stimulus and, hence, on the stimulus intensity. Using a flicker frequency of 12 Hz (at which large differences were observed between responses of WT and EAAT5^−/−^ retina), retinae of both genotypes were stimulated at stimulus intensities from the scotopic to the high mesopic range. Figure 6 shows the fraction of correctly responding cells plotted against stimulus intensity for different ganglion cell types. For ON-merge cells (Fig. 6*A*), other ON-ganglion cell types (Fig. 6*B*, pooled ON-ganglion cells except for ON-merge cells), and OFF-ganglion cells (Fig. 6*C*) at low stimulus intensities, these values were small and differences between the two mouse genotypes were not significant. At the highest intensity, the fraction of correctly responding cells was larger in WT retina than in EAAT5^−/−^ retina. The difference was statistically significant for ON- but not for OFF-ganglion cells. With higher stimulus intensity, OFF-ganglion cells failed to respond to each flash of a flicker series, probably because they received more inhibition mediated by the rod bipolar/AII pathway (Smith et al., 1986; Wässle et al., 1986; Müller et al., 1988). The failure to respond to each flash reduced their apparent flicker resolution.

**Figure 7.**
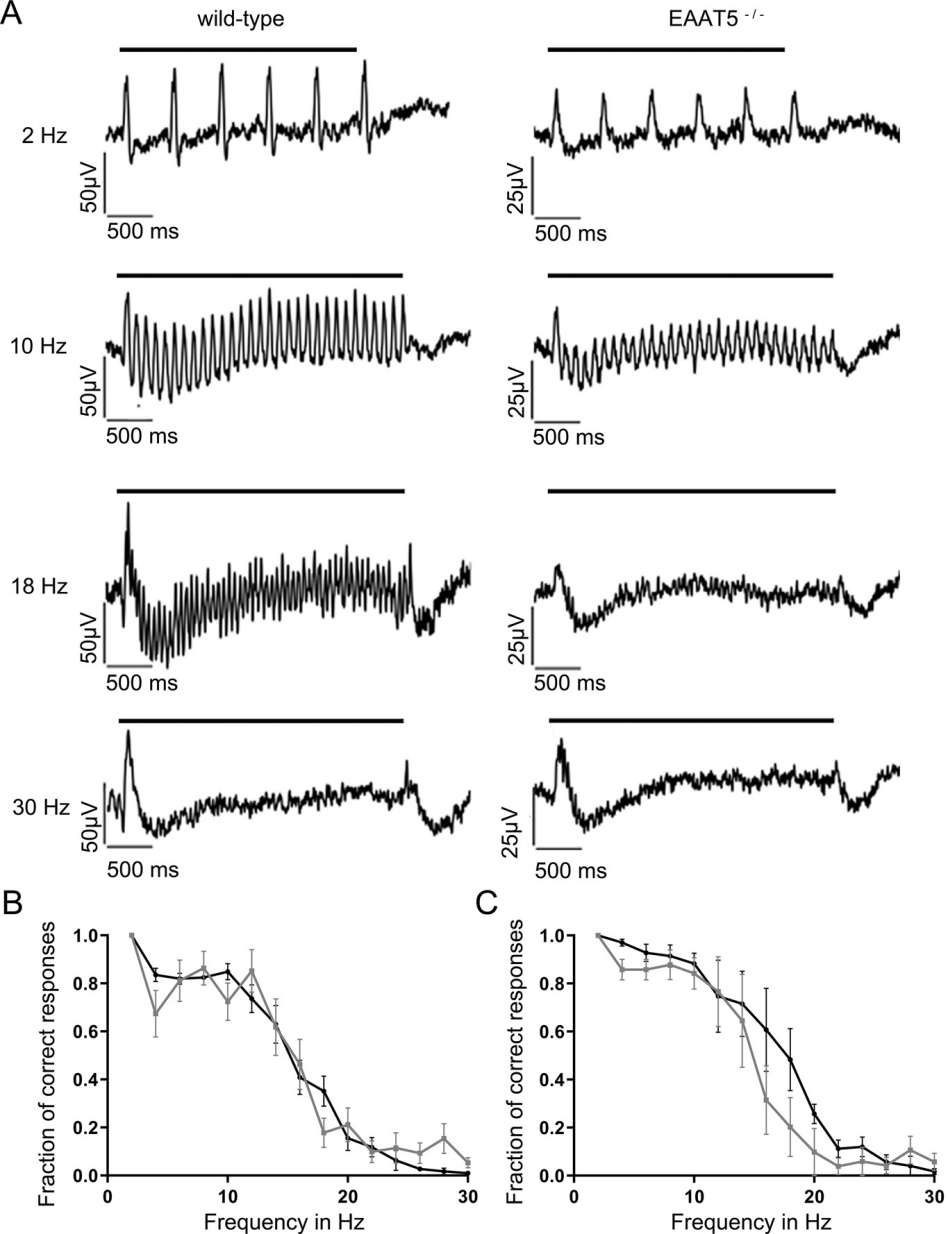
Temporal resolution of the EAAT5^−/−^ retina is not significantly impaired in the photopic range. Flicker stimuli were applied for a series of frequencies from 2 to 30 Hz. ***A***, For the 2-Hz flicker stimulus, both genotypes showed a clear response. The LFP of EAAT5^−/−^ retina (right) showed slightly impaired temporal resolution for 10- and 18-Hz flicker stimuli compared with the WT retina (left). Neither WT nor EAAT5^−/−^ retinae could resolve the 30-Hz flicker stimulus. Bars: stimulus duration. ***B***, The fraction of correct responses plotted against the stimulus frequency for stimulus intensity of 1000 activated rhodopsin molecules (rhod*/rod/flash) shows no significant reduction for EAAT5^−/−^ (gray curve, mean ± SEM of eight retinal pieces from six animals) compared with the WT (black curve, mean ± SEM of 10 retinal pieces from six animals). Two-way ANOVA with Bonferroni multiple comparisons. ***C***, The fraction of correct responses plotted against the stimulus frequency for stimulus intensity of 19,000 activated rhodopsin molecules (rhod*/rod/flash) shows a mild but not significant reduction for EAAT5^−/−^ (gray curve, mean ± SEM of eight retinal pieces from six animals) compared with the WT (black curve, mean ± SEM of 10 retinal pieces from six animals). Two-way ANOVA with Bonferroni multiple comparisons. Total stimulus duration: 3 s; individual flash duration: 20 ms; background light: 10,000 rhod*/rod/s.

Next, it was tested whether temporal resolution in the EAAT5^−/−^ was also compromised under photopic conditions. After light adaptation *in vitro* for 10 min to suppress the rod contribution and isolate cone-driven responses (Seeliger et al., 2011), the LFP waveform changed to resemble the characteristic ERG for light-adapted retinae (Fig. 7*A*). The large a-wave-like deflections were strongly reduced and the b-waves became shorter (compare Figs. 4*A* and 7*A*).

In the light-adapted state, differences in temporal resolution of the two genotypes were much smaller than under mesopic conditions. As shown in Figure 7*A*, the retinae of both genotypes could resolve the 2-Hz flicker stimulus equally well but not the 30-Hz flicker stimulus. For the WT, amplitudes of the initial and successive b-waves were very similar for 2- and 10-Hz flicker stimuli; at 18 Hz, the initial b-wave was twice as large as its successor. In the EAAT5^−/−^ retina at 10 Hz, amplitudes of the second and following b-waves were reduced by two-thirds compared with the initial b-wave; at 18 Hz, the flicker stimulus was barely resolved. Recordings were performed at stimulus intensities of 1000 (Fig. 7*B*) and 19,000 (Fig. 7*C*) isomerizations per rod and flash. While there was a tendency for lower resolution in EAAT5^−/−^ retina (gray curves) compared with WT retina (black curves), differences did not reach statistical significance under photopic conditions.

## Discussion

This study introduces a new mouse model that enabled studying the impact of EAAT5 on retinal light responses in the intact retina for the first time. The major outcome of the study is: EAAT5 is highly localized in close proximity to the glutamate release sites at photoreceptor and bipolar cell synapses. Genetic ablation of EAAT5 severely affected retinal output by compromising temporal resolution.

### EAAT5 expression

In rod bipolar cells, fluorescence staining showed bright puncta of EAAT5 at the axon terminal in retinal sections, as well as in dissociated cells. In the OPL, the situation is less clear. EAAT5 puncta in the invagination colocalized with neither the CabP28K marker of horizontal cell dendritic tips (Haverkamp and Wässle, 2000) nor with mGluR6 (on ON-bipolar cells). In the rat retina, mGluR6 localizes not to the extreme tip of ON-bipolar cell dendrites but more proximally, at 200–600 nm from the active zone of the invagination (Vardi et al., 2000). Thus, EAAT5 might localize more distally than mGluR6 on ON-bipolar cell dendrites. Unfortunately, rod bipolar cell markers (PKCα and Gα_0_) did not label dendrites distal to mGluR6, so colocalization with EAAT5 could not be tested (data not shown). Finally, EAAT5 might be expressed presynaptically in the photoreceptor membrane within the invagination. EAAT5 expression in photoreceptors was suggested previously (Eliasof and Werblin, 1993; Picaud et al., 1995; Pow and Barnett, 2000; Hasegawa et al., 2006; Wersinger et al., 2006; Lee et al., 2013). Tse et al. (2014) also reported punctate EAAT5 staining (using a commercial antibody) in the OPL of mouse retina, but did not associate these puncta with the glutamate release site. Using the same commercial antibody in double labeling experiments, 100% colocalization with the newly raised anti-EAAT5 antibody was found (data not shown). Other studies on EAAT5 have described a more diffuse signal with no clear association with the glutamate release site (e.g., labeling of the entire terminal, somata, and even the inner segments of photoreceptors; Pow and Barnett, 2000; Wersinger et al., 2006). Of course, the antibodies used in these experiments have not yet been tested on EAAT5^−/−^ retina.

### Function of EAAT5

So far, the absence of an EAAT5-specific blocker has made it impossible to distinguish the functions of this transporter from those of other EAAT isoforms in the intact retina, in particular in shaping light responses. For example, in all previous studies EAAT-mediated currents in the retina were identified using EAAT blocker DL-*threo*-β-benzyloxyaspartic acid (TBOA), which does not discriminate between EAAT isoforms. The kinetics of TBOA-blockable currents in bipolar cells (Veruki et al., 2006) were not identical to those in cells with heterologously expressed EAAT5 (Gameiro et al., 2011; Schneider et al., 2014). These differences may depend on the recording conditions or reflect differences between the recorded cell types; however, the possibility that the results of previous studies may not reflect the function of EAAT5 alone, but may also include those of other EAAT isoforms (e.g., EAAT2 in bipolar cells; Rauen et al., 1996; Harada et al., 1998) cannot be excluded.

In this study, by establishing an EAAT5 KO mouse model, functions could be specifically attributed to this EAAT isoform. EAAT5 deletion specifically affected retinal output by compromising temporal resolution. EAAT5 might improve temporal resolution via different mechanisms: (1) glutamate-gated chloride currents mediated by EAAT5 could trigger feedback inhibition; (2) by buffering glutamate or clearing glutamate from the synaptic cleft, EAAT5 would reduce the action of glutamate at postsynaptic as well as on presynaptic glutamate receptors.

In neuronal networks, diverse feedback mechanisms adjust the gain control to limit the response amplitude (thereby preventing saturation of cellular and network activity) and increase temporal resolution by shortening the responses. In the retina, presynaptic and postsynaptic feedback mechanisms control every step of signal processing. An example of presynaptic feedback is activation of HCN1 channels (hyperpolarization-activated and cyclic nucleotide-gated ion channel 1) during the hyperpolarizing light response of photoreceptors, which effectively curtails the light response (Fain et al., 1978; Knop et al., 2008; Seeliger et al., 2011). Targeted deletion of HCN1 reduced flicker resolution and led to saturation of the retinal network by rod activity (Seeliger et al., 2011). EAAT5 might mediate another presynaptic feedback mechanism at retinal glutamatergic synapses that strongly improves temporal resolution. EAAT5 was found strategically localized for this function.

### EAAT5 in rod bipolar cells

The effect of EAAT5 on temporal resolution was particularly pronounced under mesopic light conditions when both rods and cones are active but rods contribute a substantial fraction to the light response. Upon EAAT5 deletion, ON-ganglion cell spiking as well as b-waves of the LFP were strongly affected, both of which depend on rod bipolar cell activity. Characteristics of the b-wave can be affected by changing photoreceptor input to rod bipolar cells. For example, application of APB acting at mGluR6 at rod bipolar cell dendrites can abolish the b-wave (Slaughter and Miller, 1981; Massey et al., 1983). Changing signal processing within the rod bipolar cells also affects the b-wave. For example, deletion of PKCα, which is highly expressed in rod bipolar cells, affects proper activation and termination of the rod bipolar cell responses and, hence, the characteristics of the b-wave (Ruether et al., 2010; Xiong et al., 2015).

The effect of EAAT5 deletion is in perfect agreement with the postulated role of EAAT5 at rod bipolar cell terminals. First, EAAT5 was unequivocally localized in form of brightly fluorescent puncta to the output synapses in both sections and isolated rod bipolar cells. Second, EAAT5 was shown to act as glutamate-gated chloride channel in rod bipolar cells (Veruki et al., 2006; Wersinger et al., 2006; Bligard et al., 2020). Upon depolarization of the rod bipolar cell, glutamate release at the output synapse would activate not only the postsynaptic glutamate receptors but also presynaptic EAAT5, leading to chloride influx, hyperpolarization of the cell, and, consequently, reduced bipolar cell output. Indeed, chloride currents could be elicited by depolarization and were inhibited by TBOA, providing support for a postulated EAAT5-mediated feedback mechanism. Bligard et al. (2020) postulated that EAAT-mediated inhibition is important for gain control at the rod bipolar cell output synapse. Owing to the time delay needed for activation, EAAT5-mediated inhibition may preferentially affect the later stage of depolarization and, therefore, shorten rod bipolar cell output. Shorter signals would enable the transmission of higher frequencies at this synapse. Finally, flicker resolution is higher in the cone system than in the rod system. By reducing rod bipolar cell output under mesopic conditions, EAAT5-mediated feedback might shift the balance toward the cone system to enable higher flicker resolution. However, the improvement of temporal resolution might also depend on functions of EAAT5 apart from its function as a chloride channel. EAAT5 might improve glutamate buffering and reuptake in the synaptic cleft. This glutamate clearance would help to sharpen the action of glutamate at postsynaptic cells, thereby increasing temporal resolution in WT retina. In EAAT5^−/−^ retina, at low stimulus frequencies used (e.g., at 2 Hz), glutamate might diffuse from the synaptic cleft to be removed by EAAT1 and local glutamate concentration could return to baseline between individual light flashes even in the absence of EAAT5. At higher flicker frequencies, however, synaptic events might be considerably prolonged, thereby “blurring” synaptic activity and reducing temporal resolution.

Genetic ablation of EAAT5 only slightly lowered flicker resolution under photopic conditions (where the cone system dominates the light response). In electrophysiological experiments, no EAAT-like currents have been identified in mouse cone bipolar cells (Wersinger et al., 2006; Bligard et al., 2020). However, since there are at least 12 types of cone bipolar cells (for review, see Euler et al., 2014) and bipolar cells can differ substantially in their inventory of ion channels (Ivanova and Müller, 2006), the small samples of recorded cells in these studies might not have included all bipolar cell types. Some EAAT5-immunoreactive puncta were associated with cone bipolar cell terminals (Fig. 3*A*). Based on their stratification level in the IPL, some of these terminals might correspond to type five cone bipolar cells, which are ON-cone bipolar cells. EAAT5-like immunoreactivity in some cone bipolar cells was also reported by Pow and Barnett (2000) and Fyk-Kolodziej et al. (2004). Thus, while the evidence of EAAT5 expression is less compelling for cone bipolar axon terminals and EAAT5 seems to be of lesser importance for photopic vision, immunohistochemical data suggest that EAAT5 might play a role in controlling synaptic output at least in some cone bipolar cell types.

### EAAT5 in the outer retina

There is also evidence for EAAT5 expression in photoreceptor terminals (this study; see also Eliasof and Werblin, 1993; Picaud et al., 1995; Pow and Barnett, 2000; Hasegawa et al., 2006; Wersinger et al., 2006; Lee et al., 2013). Photoreceptor synapses show a complex organization and multiple feedback mechanisms at these synapses have been described. Therefore, the physiological role of EAAT5 and the effect of EAAT5 deletion in the outer retina are more difficult to interpret than in the inner retina. Although EAAT5 is considered a glutamate transporter with a low transport rate (Gameiro et al., 2011; Schneider et al., 2014), Hasegawa et al. (2006) reported that glutamate clearance at the photoreceptor synapse mediated by EAAT5 is important for shaping of light responses at the rod–rod bipolar cell synapse in mice. KO of EAAT5 in photoreceptors might lead to elevated levels of glutamate in the synaptic cleft, triggering a variety of possible effects. For example, a mGluR was reported on cone terminals that might become activated and could affect the rate of glutamate release (Van Hook et al., 2017). Depolarization of horizontal cells by increased glutamate levels would affect photoreceptor output in a complex manner. For example, feedback by horizontal cells does not only affect photoreceptor membrane potential but also causes changes in peak amplitude as well as a shift in the voltage-dependence of cone calcium channels (Verweij et al., 1996; for review, see Thoreson and Mangel, 2012; Wen and Thoreson, 2019). Finally, EAAT5 might affect photoreceptor output via its function as chloride channel. Measurements of the chloride reversal potential in salamander (Thoreson and Bryson, 2004) and mammalian cones (Szmajda and DeVries, 2011) indicate a relatively positive chloride Nernst potential, which would argue against a negative feedback. Moreover, in a substantial body of work on salamander photoreceptors, Thoreson and colleagues speculated that chloride dynamics in the photoreceptor terminal affect the activation properties of voltage-activated calcium channels (Thoreson et al., 2000, 2003; Thoreson and Bryson, 2004; Li et al., 2008). Activation of EAAT5 triggers a chloride conductance that might affect the chloride concentration in the small terminal, and hence, photoreceptor output. This delicate interaction would be suited to fine tune photoreceptor output and must be determined in future studies.

In summary, the mechanisms by which EAAT5 could affect information transfer in outer retinal synapses are quite diverse and more experiments, in particular involving single-cell recordings or paired recordings of photoreceptors, horizontal cells, and bipolar cells are required to address this question. This is well beyond the scope of the present study that focused on the introduction and characterization of a new mouse model to study EAAT5 function, thereby overcoming the lack of an EAAT5-specific inhibitor. The mouse model enabled to demonstrate for the first time the role of EAAT5 in the intact retina. EAAT5 considerably increases temporal resolution of the retina. EAAT5 was found highly localized to glutamatergic ribbon synapses in both synaptic layers, consistent with a role in shaping the output of glutamatergic cells in the retina. Interestingly, EAAT5 is also expressed at the ribbon synapses of vestibular hair cells (Dalet et al., 2012) but not at the calyx of Held (Palmer et al., 2003), a well-studied conventional glutamatergic synapse. Synaptic activity in ribbon synapses is based on graded potentials of the presynaptic cell and is typically associated with much higher, sustained vesicular release compared with conventional more transient synapses. It is, therefore, tempting to speculate that EAAT5-mediated feedback triggered by glutamate release might be a common mechanism to regulate synaptic output at ribbon synapses.

## Synthesis

Reviewing Editor: Siegrid Löwel, University of Goettingen Decisions are customarily a result of the Reviewing Editor and the peer reviewers coming together and discussing their recommendations until a consensus is reached. When revisions are invited, a fact-based synthesis statement explaining their decision and outlining what is needed to prepare a revision will be listed below. The following reviewer(s) agreed to reveal their identity: NONE. Note: If this manuscript was transferred from JNeurosci and a decision was made to accept the manuscript without peer review, a brief statement to this effect will instead be what is listed below.

The authors have adequately addressed concerns raised in a previous review. The presentation and readability of the paper, as well as the Discussion have been substantially improved.
